# Hematologic and immunological characteristics of Henoch-Schönlein purpura in rat and rabbit models induced with ovalbumin based on type III hypersensitivity

**DOI:** 10.1038/srep08862

**Published:** 2015-03-09

**Authors:** Yanhong Li, Xiaochun Feng, Lan Huang, Hua Zhu, Yanfeng Xu, Xiaolong Sui, Yuhuan Xu, Yunlin Han, Chuan Qin

**Affiliations:** 1 Key Laboratory of Human Disease Comparative Medicine, Ministry of Health, Institute of Medical Laboratory Animal Science, Chinese Academy of Medical Sciences; Key Laboratory of Human Disease Animal Models, State Administration of Traditional Chinese Medicine, Peking Union Medicine College, Beijing 100021, China; 2 Affiliated Hospital of Changchun University of Traditional Chinese Medicine, Changchun 130021

## Abstract

Henoch-Schönlein purpura (HSP) is a common systemic vasculitis in children. Animal models of HSP are needed to better understand the mechanism of HSP. Here, we investigated hematologic and immunologic profiles in HSP rat and rabbit models. Models were established with ovalbumin (OVA) based on type III hypersensitivity. During the acute phase, the models exhibited varying degrees of cutaneous purpura, joint inflammatory response, gastrointestinal bleeding, glomerular capsule protein exudation, vascular dilatation, and increased IgA expression and immune complex deposition. Twenty four hours after antigen challenge, compared with the controls, the models showed a significantly increased white blood cell count and granulocytes count and percentage, decreased number and percentage of lymphocytes, no change in platelet concentration, significantly increased serum IL-4 and TNF-α levels, and decreased CD4^+^ T cell, CD4/CD8 ratio, and C3 and C4 levels. Compared with the hematologic and immunologic profiles in pediatric HSP patients, the rat and rabbit HSP models can mimic pediatric HSP characteristics. Our studies provide two useful animal models for further investigations of the pathogenesis, diagnosis, drug screening and treatment of HSP.

Henoch-Schönlein purpura (HSP), also known as anaphylactoid purpura, is a common systemic vasculitis in children. The annual incidence of HSP may vary from 10 to 22/100,000 individuals depending on the region and season studied^1^,^2^,^3^,^4^. HSP is characterized by deposition of IgA immune complexes (IC). The exact etiology of HSP is unknown, but exposure to antigens such as infectious agents, vaccines, and drugs may stimulate the immunological changes^4^,^5^.

Diagnosis of HSP is based on the presence of non-thrombocytopenic purpuras, which typically appear on the legs and buttocks, but may also be seen on the arms, face, and trunk. Other clinical symptoms of HSP include joint involvement with pain, edema, abdominal pain, gastrointestinal hemorrhage, and renal involvement with hematuria or proteinuria^6^. HSP is considered to be an immune-mediated specific disease. During the acute stage, complexes of IgA and complement C3 are deposited in the walls of arterioles, capillaries, and venules; serum levels of proinflammatory cytokines increase and inflammatory cell infiltration of peripheral vessels occurs^6^. In the majority of cases HSP is a self-limiting disease and treatment is supportive. However, HSP has a high rate of recurrence, and some patients can progress to HSP nephritis (HSPN), which can result in renal failure^7^.

The pathogenesis of HSP is complex and has not been fully understood. Animals are excellent models of many physiological processes and diseases in humans; however, reports on animal models of HSP are scarce. The objective of this study was to establish HSP rat and rabbit models with ovalbumin (OVA) to induce type III hypersensitivity. The hematologic and immunologic profiles of HSP animal models were compared with pediatric HSP patients, and the immunological pathogenesis of HSP disease was further investigated.

## Results

### Clinical features of the skin in HSP rats and rabbits

**Figure 1:**
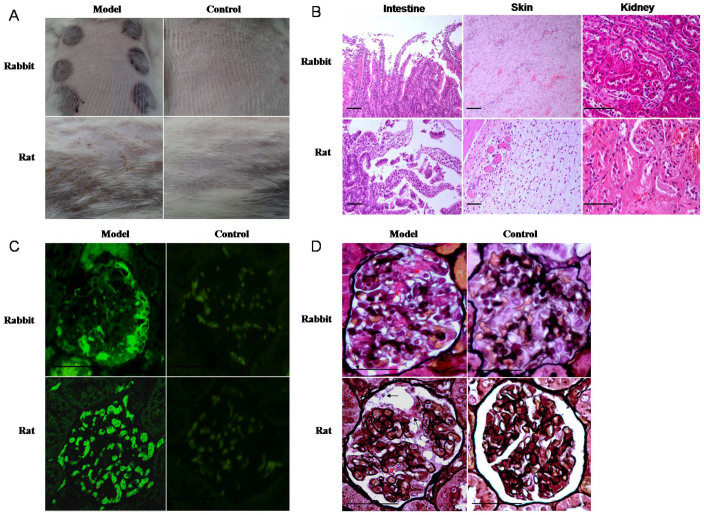
External characteristics and pathological changes in HSP rat and rabbit models (A) The skin systems of rabbit and rat in models and controls. (B) Pathological changes in HSP rabbit models: shedding of the intestinal villus epithelium, dermal hemangiectasis, hyperemia and hemorrhage, glomerular capsule cavity effusion, and protein casts in the tubular lumens; pathological changes in HSP rat models: Shedding of the intestinal villus epithelium, edema and vasodilation in the dermis, glomerular capsule cavity effusion, and protein casts in the tubular lumens. (C) IgA IC positive deposition (green arrow) in the glomerulus of the HSP rat and rabbit model, and negative in the controls. (D) Glomerular capsule cavity effusion (black arrow) and IC deposition (red arrow) in glomerular mesangial cells of the HSP rat and rabbit model. n = 10–18. Bar = 100 μm.

Six to eight hours after antigen challenge, the skin of HSP rats and rabbits developed hemorrhagic spots; 14–18 h after antigen challenge, the number and size of the hemorrhagic spots gradually increased. 40% (4/10) of the HSP rats developed isolated hemorrhagic spots with a generalized distribution (1–2 mm in diameter), and 85% of HSP rabbits (24/28) developed hemorrhagic spots (1 cm in diameter) surrounded by redness and swelling in a localized distribution. Some hemorrhagic spots were fused and/or had exudates. 48 h after antigen challenge, secondary changes in the hemorrhagic spots in both HSP rats and rabbits included the development of scabs and necrosis. Symptoms showed gradual improvement and marked improvement two and three weeks after antigen challenge, respectively (Fig. 1A).

### Pathological features of HSP rats and rabbits

Hematoxylin and eosin staining of tissue samples from several HSP rats and rabbits exhibited different degrees of inflammatory response. The skin showed subcutaneous hemorrhage, dermal edema, hemangiectasis and hyperemia, hemorrhage, and inflammatory cell infiltration. The kidney showed glomerular capsule protein exudation, vascular dilatation, and congestion. The limb joints showed cavity congestion, connective tissue necrosis, and inflammatory cell infiltration. The gastric mucosa showed hemorrhage, epithelial cell necrosis and shedding, and small intestinal villus angiectasis hyperemia. The lung and liver showed congestion and focal inflammatory cell infiltration. A meningeal inflammatory reaction included angiectasis hyperemia and inflammatory cell infiltration (Fig. 1B). IgA expression and IC deposition increased in the glomerular mesangial cells and basement membrane in both HSP rats and rabbits compared with controls (Fig. 1C, D).

### Hematologic profile in HSP animal models and pediatric HSP patients

**Figure 2:**
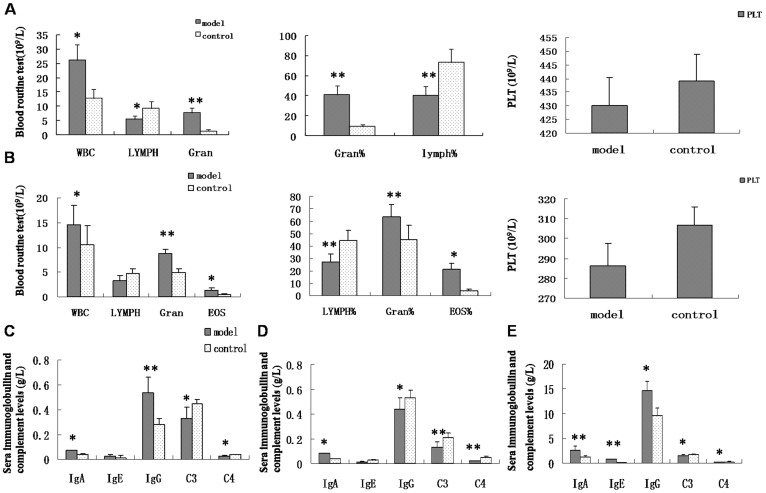
Routine blood, serum immunoglobulin and complement levels in animals and pediatric patients. (A, B) Routine blood analyses in model rats and rabbits, respectively. WBC, Gran and Gran% increased, while LYMPH% decreased and there was no significant change in PLT in both animals compared to the controls. LYMPH decreased in HSP rats, while EOS and EOS% increased in HSP rabbits. (C, D, E) The changes of serum immunoglobulin and complement level in HSP rats, rabbits, and pediatric patients, respectively. IgA level increased, while C3 and C4 levels decreased in HSP animals and patients compared to controls. IgG levels increased in HSP rats and patients, and decreased in HSP rabbits. IgE levels increased in HSP patients and did not change in the model animals. n = 10–18. * *P* < 0.05 and **P < 0.01 vs. controls.

During the acute phase following antigen challenge, the white blood cells (WBC) count (*P* < 0.05) and the number (Gran; *P* < 0.01) and percentage (Gran%; *P* < 0.01) of granulocytes significantly increased in HSP rats compared with controls. In addition, the number (LYMPH; *P* < 0.05) and percentage (LYMPH%; *P* < 0.01) of lymphocytes significantly decreased, and there was no change in the number of platelets (PLT) (Fig. 2A). In HSP rabbits, the WBC count (*P* < 0.05), Gran (*P* < 0.01), Gran% (*P* < 0.01), and the number (EOS; *P* < 0.05) and percentage (EOS%; *P* < 0.05) of eosinophils significantly increased, while the LYMPH% (*P* < 0.01) significantly decreased, and there was no change in PLT (Fig. 2B). In pediatric HSP patients, the WBC count, Gran, and the number (MONO) and percentage (MONO%) of monocytes significantly increased (*P* < 0.05) compared with controls, and increased beyond the normal reference range in 33.3% (4/12), 25% (3/12),41.6% (5/12), and 33.3% (4/12) of patients, respectively. LYMPH% (*P* < 0.05) significantly decreased compared with controls, and lower than the normal reference range in 25% (3/12) of patients, while there was no difference in PLT counts (Table 1).

### Serum immunoglobulin and complement levels in HSP animal models and pediatric HSP patients

During the acute phase following antigen challenge, serum IgA (0.073 ± 0.005 *vs.* 0.044 ± 0.005 g/L) (*P* < 0.05) and IgG (0.54 ± 0.082 *vs.* 0.28 ± 0.049 g) (*P* < 0.01) levels significantly increased in HSP rats compared with controls. The C3 (0.33 ± 0.09 *vs*. 0.45 ± 0.03 g/L) (*P* < 0.05) and C4 (0.027 ± 0.01 *vs.* 0.038 ± 0.006 g/L) (*P* < 0.05) levels significantly decreased and there was no change in serum IgE levels (0.03 ± 0.009 *vs*. 0.025 ± 0.003 g/L) in HSP rats compared with controls (Fig. 2C). In HSP rabbits, the serum IgA levels (0.084 ± 0.0005 *vs.* 0.04 ± 0.00003 g/L) (*P* < 0.05) significantly increased compared to the controls, while the IgG (0.44 ± 0.09 *vs.* 0.53 ± 0.06 g/L) (*P* < 0.05), C3 (0.13 ± 0.048 *vs*. 0.21 ± 0.035 g/L) (*P* < 0.01), and C4 (0.02 ± 0.004 *vs*. 0.05 ± 0.010 g/L) (*P* < 0.01) levels significantly decreased, and there was no change in serum IgE levels (0.011 ± 0.0067 *vs*. 0.029 ± 0.0057 g/L) compared to the controls (Fig. 2D). In pediatric HSP patients, the serum IgA (2.61 ± 0.85 *vs*. 1.2 ± 0.31 g/L) (*P* < 0.01), IgE (0.83 ± 0.063 *vs*. 0.079 ± 0.003 g/L) (*P* < 0.01), and IgG (14.65 ± 1.8 *vs*. 9.6 ± 1.51 g/L) (*P* < 0.05) levels were significantly increased compared to the controls, while the C3 (1.45 ± 0.25 *vs*. 1.73 ± 0.16 g/L) (*P* < 0.05) and C4 (0.19 ± 0.05 *vs*. 0.28 ± 0.09 g/L) (*P* < 0.05) levels significantly decreased (Fig. 2E).

### Peripheral blood T lymphocyte subsets and cytokine levels in HSP animal models and pediatric HSP patients

**Figure 3:**
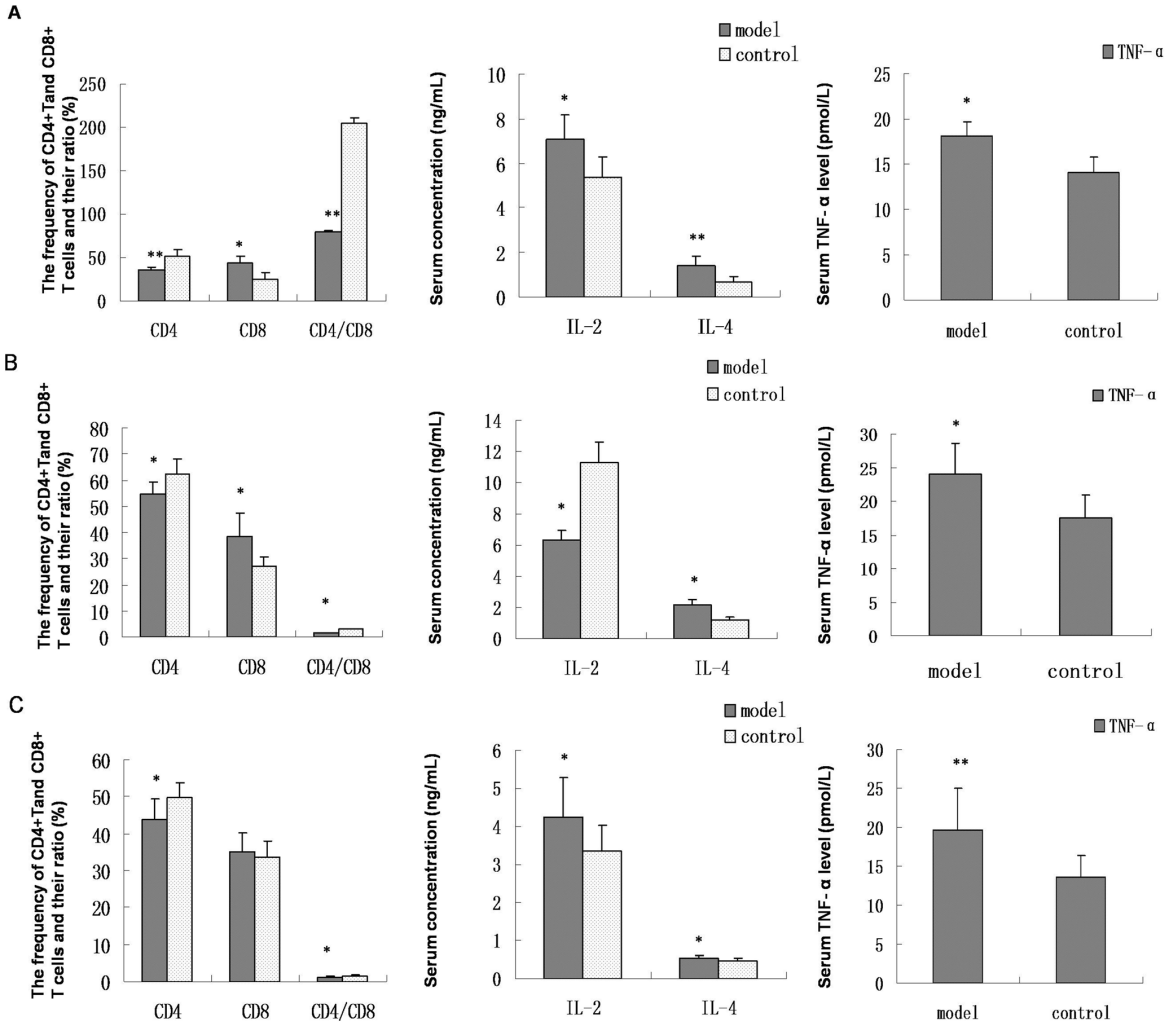
Peripheral blood T lymphocyte subsets and cytokine levels in HSP animals and pediatric patients. (A, B, C) Changes in the frequency of peripheral blood CD4+ and CD8+ T cells, ratio of CD4/CD8, and serum IL-2, IL-4, and TNF-α levels in HSP rats, rabbits, and patients, respectively. The frequency of CD4+ T cells and the ratio of CD4/CD8 decreased, and IL-4 and TNF-α levels increased in HSP animal models and patients compared to the controls. The frequency of CD8+ T cells increased in the model animals and there was no difference in HSP patients. IL-2 levels decreased in the model rabbits, and increased in HSP rats and patients. n = 10–18. * *P* < 0.05 and **P < 0.01 vs. controls.

During the acute phase following antigen challenge, in HSP animals and pediatric patients, compared to the controls, the frequency of CD4+ T cells significantly decreased (*P* < 0.05), the frequency of CD8+ T cells increased (rats and rabbits: *P* < 0.05; pediatric patients: *P* > 0.05), and the ratio of CD4/CD8 significantly decreased (rats: *P* < 0.01; rabbits and pediatric patients: *P* < 0.05). Serum IL-4 (*P* < 0.05) and TNF-α (animals: *P* < 0.05; pediatric patients: *P* < 0.01) levels significantly increased. In HSP rats and pediatric patients, the IL-2 level (*P* < 0.05) increased, while in HSP rabbits, the IL-2 level (*P* < 0.05) decreased (Fig. 3A, B, C).

## Discussion

Reports describing successfully established animal models of HSP are scarce. This study compares the hematologic and immunologic profiles of HSP rat and rabbit models with HSP pediatric patients. Han^10^ developed an HSP mouse model, which is referred to as a model of IgA nephropathy; however, only 3 of 20 animals developed skin purpura. Zhang^11^ described an HSP rabbit model, but only investigated skin and kidney manifestations. In the current study, rat and rabbit models were established that focused on the etiology, pathogenesis, and pathological changes associated with HSP disease. Initially, individual differences in sensitivity to allergens due to genetic susceptibility among animals were eliminated with Traditional Chinese Medicine. Three traditional Chinese medicines were administered to the model animals to induce a thermal effect to cause dilation of blood vessels, increase susceptibility to allergic reactions, and ensure the animals had a consistent allergic constitution^8^. Subsequently, animal models of allergic systemic vasculitis were established based on type III hypersensitivity.

Both animal models developed hemorrhagic spots; however, the size and the distribution of the hemorrhagic spots varied (Fig. 1A). The time that both animal models began to develop symptoms was the same. All pediatric HSP patients developed a non-pruritic rash, which progressed from an erythematous papular rash or urticaria to petechiae, purpura, and obvious ecchymoses^12^. The ecchymoses changed color from red to purple and subsequently faded^13^. Both animal models exhibited histopathological changes in their joints, gastrointestinal tracts, and glomeruli. Pathological changes in pediatric HSP patients include joint involvement with pain, edema, or arthritis in 80% of cases, and abdominal pain in 60–65% cases, with gastrointestinal hemorrhage in 30% of cases^2^,^3^,^14^. Kidney damage is the most serious sequelae and a major cause of mortality^15^. Taken together, these data indicate that our HSP animal models can mimic human HSP disease in terms of symptoms and histopathological changes.

During the acute phase, in HSP animals and pediatric patients, serum IgA levels increased and kidney-deposited IgA levels in the animal models also increased. However, the higher serum IgA levels are still less than 1/10 of the IgG levels in the body^16^. In accordance with our data, evidence suggests that serum IgA levels are increased in over 50% of HSP patients^10^,^17^,^18^ and that active HSP patients have 2-fold more IgA secreting cells than controls^19^. The extent of the changes in serum IgG and IgE levels in our HSP model animals and pediatric patents were different, but C3 and C4 levels were all decreased compared with controls. Previous data describing serum IgG and IgE levels in HSP patients are not entirely consistent. Various studies suggest IgG levels may decrease, increase, or show no change^20^,^21^,^22^. In agreement with these findings, Levy et al^23^ reported that serum C4 level was decreased in 18.7% of HSP patients and other studies demonstrated that serum C3 and C4 levels were decreased in 8% of HSP patients^24^,^25^. Several publications show that serum C3 and C4 levels in HSP patients are increased in areas of tissue damage, but serum levels have no relation to disease severity^24^,^25^,^26^. These observations indicate that IgA is the main immunoglobulin responsible for the pathogenesis of HSP.

The changes in parameters measured in routine blood tests were variable among animal models and patients; however, the data reflected the presence of an immunological disorder to a certain extent. Alterations in the frequency of CD4+ and CD8+ T cells, their ratio, and serum TNF-α and IL-4 levels in HSP animals were in accordance with HSP patients, while patterns of IL-2 levels were different. Previous reports describe changes in peripheral blood levels of CD4+ T, CD8+ T cells, CD4/CD8, TNF-α, and IL-4 in pediatric HSP patients that are consistent with our findings^27^,^28^,^29^. Other studies indicate that serum IL-2 levels are decreased^20^,^28^ and, at the acute stage, IL-2 receptor number and function are increased and enhanced, respectively, in association with disease activity^28^. Our results show variability between the IL-2 levels in HSP model animals and pediatric patients, possibly because they were experiencing different levels of disease activity.

In conclusion, the symptoms and pathological and immunological changes in HSP rat and rabbit models were consistent with characteristics in pediatric HSP patients. When developing animal models of HSP, it is important to choose animals that are easy to manipulate and observe, with skin that is sensitive to stimulation. In addition, the selection of drug dose should be moderate to avoid anaphylactic shock and experimental results should mimic human disease and have high repeatability. In accordance with these requirements, the rabbit model may be a useful and novel tool for the study of HSP. The development of the rat as an additional animal model of HSP with different characteristics may provide more choices and alternative approaches for the study of pathogenesis of HSP disease, as well as drug screening and evaluation.

## Methods

### Materials

Animals (rats and rabbits) and pediatric HSP patients are described below. Animal experiments were conducted according to the guidelines of animal welfare of the World Organization for Animal Health, and approved by the Institute of Animal Use and Care Committee of the Institute of Laboratory Animal Science, Peking Union Medical College (permit number: ILAS-PL-2012-005).

Human clinical studies were approved by the National Ethics Committee and were performed in accordance with the ethical standards of the World Medical Association's Declaration of Helsinki. Written informed consent was obtained from the parents of all patients enrolled in the study and patient anonymity was preserved.

### Animals

Sprague Dawley rats were purchased from Beijing Wei Tong Li Hua Experimental Animal Technology Co., Ltd. (SCXK [Beijing] 2012-0001) and Japanese big-ear white rabbits were purchased from Beijing Fang Yuan Marginal Farms (SCXK [Beijing] 2009-0014). Rats (n = 22; 4 weeks old; 75–95 g; 1:1 male: female) and rabbits (n = 28; 1.5 month old; 1.2–1.4 kg; 1:1 male: female) were divided into an HSP model group (rats: n = 11; rabbits: n = 18) and a control group (rats: n = 11; rabbits: n = 10). Animals were housed in specific pathogen-free (SPF) animal house facilities at the Institute of Medical Laboratory Animal Science, Chinese Academy of Medical Sciences (SYXK [Beijing] 2011-0022).

### Pediatric patient recruitment

Pediatric HSP and control patients (4–12 years old) were recruited from the Affiliated Hospital of Changchun University of Traditional Chinese Medicine. HSP patients (n = 12) were included in the study if they were untreated and manifested obvious symmetrical purpura on the skin of the lower extremities; 5 patients had detectable hematuria and proteinuria. Control (n = 10) patients were healthy children of a similar age attending a routine check-up at the hospital.

### Establishment of HSP rat and rabbit models

Individual differences in sensitivity to allergens due to genetic susceptibility among animals were eliminated with Traditional Chinese Medicine^8^. Briefly, ginger, long pepper, and pepper decoction purchased from the Beijing Dongzhimen Hospital at the University of Traditional Chinese Medicine were mixed in a 1:1:1 ratio in an aqueous solution (15 g/100 mL) and incubated in a water bath at 60°C for 30 minutes until dissolved, then filtered and stored at 4°C. Model group animals were administered the stored compound solution (0.125 g/kg) each day; animals in the control groups received equal amounts of physiological saline^8^. Following three weeks of dosing, the model group animals received ovalbumin (OVA) (F5503; Sigma) emulsified solution (10 mg/kg), consisting of OVA solution (20 mg/ml) and Freund's complete adjuvant (F5881; Sigma) mixed in a 1:1 ratio, by intraperitoneal injection once per week for three weeks. Subsequently, HSP rats each received OVA physiological saline liquor (10 mg/mL; 0.25 mL) by tail vein injection; HSP rabbits each received OVA physiological saline liquor (10 mg/mL; 0.5 mL) by ear vein injection. HSP animals each also received OVA physiological saline liquor (0.3%; 1 mL; 5 injection points) through an intradermal injection. Animals in the control groups received equal amounts of physiological saline.

### Hematologic and immunologic profile of HSP rat and rabbit models and pediatric patients

Blood samples were collected with EDTA-K2 evacuated tubes (BD Vacutainer, USA) from animals within 24 hours of antigen challenge and from HSP patients during a hospital visit to detect routine histopathology by an automatic blood cell analyzer (Rayto RT-7600S, USA) and T lymphocyte subset levels. Blood samples were collected with normal serum tubes (BD Vacutainer, USA) from animals and patients to detect serum IgA, IgE, IgG, C3, and C4 levels by immunonephelometry (Hitachi 7080, Japan) and the cytokines IL-2, IL-4 and TNF-α.

In rats, serum IL-2 and TNF-α levels were detected by radioimmunoassay (XH-6020γ- Immune counter, China); serum IL-4 levels were detected by enzyme-linked immunosorbent assay (ELISA) (microplate reader ST-360, China); frequency of CD4+ T cells (85-11-0040-82, eBioscience) and CD8+ T cells (85-11-0084-82, eBioscience) cells in serum were detected by flow cytometry (FACSCalibur, BD, USA).

In rabbits, serum IL-2 (CSB-E08745Rb, Cusabio), IL-4 (CSB-E06902Rb, Cusabio), and TNF-α (CSB-E06998Rb, Cusabio) levels, and concentrations of CD3+ (QT-T98065, Qiyibio), CD4+ (CSB-E12791Rb, Cusabio) and CD8+ (CSB-E12790Rb, Cusabio) T cell markers were detected by ELISA.

In pediatric patients, serum IL-2 (HE0064, Huayibio), TNF-α (BH1105, Huayibio), and IL-4 (HE0060, Huayibio) levels and concentrations of CD3+ (CSB-E12706h, Cusabio), CD4+ (BH1061, HuayiBio) and CD8+ (KA0133, Abnova) T cell markers were detected by ELISA.

Three weeks after antigen challenge, histopathologic analysis of animal tissues was performed using haematoxylin-eosin staining, immunofluorescence staining with FITC-IgA (anti-rabbit IgA: ab97189, anti-rat IgA: ab97184, Abcam), and IC detection by Gomori methenamine silver –Masson improved staining^9^.

### Statistical analysis

Statistical analyses were performed using SPSS 16.0 software (SPSS Inc., Chicago, IL, USA). Data are expressed as mean ± SD. Between-group differences were assessed with the Mann-Whitney U test. *P* < 0.05 was considered statistically significant. The frequency of CD4+ and CD8+ T cells and CD4/CD8 was calculated directly from flow cytometry (rats) or as a percentage based on the concentration of cell markers detected by ELISA (rabbits and pediatric patients). After staining, tissue slices were analyzed with the Aperio slice scanner (Aperio Technologies, Inc. Vista, CA 92081, USA); results were evaluated using Image-pro-plus software (Media cybernetics, Inc. IPP 5.0, USA).

## Author Contributions

C.Q., X.F. and Y.L. designed the experiments. Y.L., X.S., H.Z. and L.H. performed the animal experiments; Y.X., Y.H. and Y.X. performed the paraffin sectioning and pathological diagnosis; Y.L. performed other experiments, analyzed the data and wrote the manuscript. All authors reviewed the manuscript.

## Figures and Tables

**Table 1:** Hematologic profile in pediatric Henoch- Schönlein purpura (HSP) patients

Group	WBC	LYM	LYM%	Gran	Gran%	MONO	MONO%	PLT
Reference range	(4–10) × 10^9^/L	(0.8–4) × 10^9^/L	(20–40)%	(2–7.7) × 10^9^/L	(50–70)%	(0.12–0.8) × 10^9^/L	(3–8)%	(100–300) × 10^9^/L
Patients (n = 12)	9.05 ± 0.97*	2.14 ± 0.38	25.05 ± 2.23*	6.79 ± 0.91*	67.50 ± 4.75	0.77 ± 0.08*	7.97 ± 1.81*	260 ± 10.19
Control (n = 10)	6.58 ± 1.03	3.10 ± 0.56	32.63 ± 3.1	4.74 ± 0.70	58.58 ± 3.91	0.51 ± 0.10	5.99 ± 0.98	276 ± 13.68

Note: WBC: white blood cell count; LYM: number of lymphocytes; LYM%: percentage of lymphocytes; Gran: number of granulocytes; Gran% percentage of granulocytes; MONO: number of monocytes; MONO%: percentage of monocytes; PLT: platelet.

*P < 0.05, compared with the control.
